# Evaluation of a Common Internal Standard Material to Reduce Inter-Laboratory Variation and Ensure the Quality, Safety and Efficacy of Expanded Newborn Screening Results When Using Flow Injection Analysis Tandem Mass Spectrometry with Internal Calibration

**DOI:** 10.3390/ijns6040092

**Published:** 2020-11-19

**Authors:** Rachel S. Carling, Catharine John, Philippa Goddard, Caroline Griffith, Simon Cowen, Christopher Hopley, Stuart J. Moat

**Affiliations:** 1 Biochemical Sciences, Viapath, Guys & St Thomas’ NHSFT, London SE1 7EH, UK; kate.john@viapath.co.uk; 2 GKT School Medical Education, Kings College London, London SE1 1UL, UK; 3 Newborn Screening & Biochemical Genetics, Birmingham Children’s Hospital, Birmingham B4, UK; Philippa.goddard@nhs.net; 4 Specialist Laboratory Medicine, Leeds Teaching Hospitals NHS Trust, Leeds LS9 7TF, UK; Caroline.griffith@nhs.net; 5 LGC, Teddington, Middlesex TW11 0LY, UK; Simon.cowen@lgcgroup.com (S.C.); Christopher.hopley@lgcgroup.com (C.H.); 6 Department of Medical Biochemistry, Immunology & Toxicology, University Hospital Wales, Cardiff CF14 4XW, UK; stuart.moat@wales.nhs.uk; 7 School of Medicine, Cardiff University, University Hospital Wales, Cardiff CF14 4XW, UK

**Keywords:** expanded screening, internal calibration, flow injection analysis tandem mass spectrometry, dried blood spots, inter-laboratory variation, isotope dilution

## Abstract

In 2015, the newborn screening (NBS) programmes in England and Wales were expanded to include four additional disorders: Classical Homocystinuria, Isovaleric Acidemia, Glutaric Aciduria Type 1 and Maple Syrup Urine Disease, bringing the total number of analytes quantified to eight: phenylalanine, tyrosine, leucine, methionine, isovalerylcarnitine, glutarylcarnitine, octanoylcarnitine and decanoylcarnitine. Post-implementation, population data monitoring showed that inter-laboratory variation was greater than expected, with 90th centiles varying from 17% to 59%. We evaluated the effect of stable isotope internal standard (IS) used for quantitation on inter-laboratory variation. Four laboratories analysed routine screening samples (*n* > 101,820) using a common IS. Inter-laboratory variation was determined for the eight analytes and compared with results obtained using an in-house common IS (*n* > 102,194). A linear mixed-effects model was fitted to the data. Using a common IS mix reduced the inter-laboratory variation significantly (*p* < 0.05) for five analytes. For three analytes, the lack of significance was explained by use of individual laboratory “calibration factors”. For screening programmes where laboratories adhere to single analyte cut-off values (COVs), it is important that inter-laboratory variation is minimised, primarily to prevent false positive results. Whilst the use of a common IS helps achieve this, it is evident that instrument set-up also contributes to inter-laboratory variation.

## 1. Introduction

In January 2015, the newborn screening (NBS) programmes in England and Wales were expanded to include four additional disorders, Classical Homocystinuria (HCU), Glutaric Aciduria type 1 (GA1), Isovaleric Acidemia (IVA) and Maple Syrup Urine Disease (MSUD). Together with Medium Chain Acyl CoA Dehydrogenase Deficiency (MCADD) and Phenylketonuria (PKU), these six disorders are collectively referred to as expanded NBS. Detection of these disorders is made by the simultaneous measurement of eight analytes on a single dried blood spot (DBS) sample by flow injection analysis tandem mass spectrometry (FIA–MS/MS). The analytes in question are total leucine (leucine + isoleucine + alloisoleucine), methionine, phenylalanine, isovalerylcarnitine (C5), glutarylcarnitine (C5DC), octanoylcarnitine (C8), tyrosine and decanoylcarnitine (C10); the latter two being utilized as secondary markers in the screening algorithms, providing further discrimination for PKU and MCADD, respectively [1].

In the UK, all laboratories adhere to nationally agreed protocols. These protocols are based on single analyte cut-off values (COVs) with disease status being suggested if the measured analyte is above the COV. A number of alternative approaches to single analyte COV have been described, e.g., floating COVs, multiple of the median (MoM), Collaborative Laboratory Integrated Reports (CLIR) functionalities [2,3,4], however, these are not permitted in England and Wales and, as such, intra-laboratory imprecision at the COV and inter-laboratory variation are both important performance parameters.

To ensure the quality and efficacy of the expanded NBS programme, in addition to individual laboratories undertaking routine internal quality control (IQC) and external quality assessment procedures, the population data were monitored centrally on a monthly basis [5]. Data were reviewed by individual instrument, individual laboratory and all laboratories. Between January and September 2015, a total of 471,589 specimens were analysed and the mean 90th centile (range) for each analyte across the 14 laboratories were: total leucine; 244 µmol/L (203–279), methionine; 29 µmol/L (21–32), phenylalanine; 74 µmol/L (64–88), C5; 0.19 µmol/L (0.16–0.24), C5DC; 0.15 µmol/L (0.12–0.22), C8; 0.07 µmol/L (0.06–0.07), tyrosine; 148 µmol/L (110–169) and C10; 0.12 µmol/L (0.07–0.17). Comparing the 90th centiles from each laboratory provided a convenient way to assess analytical performance, enabling estimation of an individual laboratory’s bias relative to the mean and highlighting that the inter-laboratory variation was greater than expected for all analytes.

The 90th centile population data were well removed from the COV for most analytes. As multiples of the mean 90th centile, COVs are 2.5, 1.7, 3.2, 10.5, 4.7, and 7.1 for total leucine, methionine, phenylalanine, C5, C5DC and C8, respectively. Whilst the risk of a false negative was considered minimal, there was concern over the potential for false positive results as only the HCU protocol incorporates second line testing and the decision was made to investigate inter-laboratory variation further. Three levels of commercial DBS IQC material were distributed to each laboratory and analysed daily for six months, providing *n* = 2214 sets of results. These data were used to estimate the uncertainty of measurement (UoM) for each analyte where UoM = intermediate imprecision = 1.96 × SD. The UoM, taken from the IQC material at the concentration closest to the COV for each analyte, was total leucine; 409 ± 94 µmol/L, methionine; 80 ± 20 µmol/L, phenylalanine; 158 ± 26 µmol/L, C5; 2.32 ± 0.56 µmol/L, C5DC; 0.46 ± 0.14 µmol/L, C8; 0.57 ± 0.1 µmol/L, tyrosine; 194 ± 40 µmol/L and C10; 0.65 ± 0.24 µmol/L. Commercial DBS IQC material can be considered as the “ideal” DBS sample as it is not subject to the pre-analytical variables that patient samples are haematocrit; i.e., spot size; spot quality [6]. Additionally, sufficient IQC material had been provided to each laboratory to ensure the location of the sub-punch was consistent so to determine the mean (range) UoM to be ± 23.8% (16 to 36) suggested that the variability in measurement was predominantly analytical in origin.

The laboratories in England (*n* = 13) and Wales (*n* = 1) essentially use identical methodology for expanded NBS, i.e., underivatised FIA–MS/MS. A fixed diameter disc (3.2 mm) is punched from the DBS specimen into a 96-well plate prior to addition of a methanolic solution containing stable isotope internal standard (IS). The solution is agitated to extract the DBS analytes and then introduced into the mass spectrometer by flow injection. The laboratory protocol [1] also specifies the mass transitions to be monitored by selective reaction monitoring (SRM) and that analytes are quantified by internal calibration. However, a questionnaire distributed to all laboratories in December 2016 [7] revealed that a number of key methodological differences existed between laboratories: 11/14 laboratories use in-house reagents, 3/14 use commercially available kits (Chromsystems MassChrom^®^ Amino Acids and Acylcarnitines from Dried Blood—LC–MS/MS and Perkin Elmer NeoBase™ Non-Derivatised MSMS); variable flow profiles are used; instrument set up and tuning is unique to each laboratory; “calibration factors” are routinely used in 11/14 laboratories. These factors are applied to individual analytes and range from 0.74 to 1.64. In many cases, a laboratory with two tandem mass spectrometers would have instrument specific factors for a given analyte. For example, phenylalanine had factors of 0.90 and 0.80 in one laboratory, C5 had factors of 1.05 and 1.25 in another laboratory [7].The evidence base for these factors was often unclear; some laboratories were aligning to historic population data, some to the Centre for Disease Control and Prevention Newborn Screening Quality Assurance Program (CDC NSQAP) and others to the UK Birmingham Quality Newborn Screening National External Quality Assessment Scheme (UK NEQAS). The latter two approaches are not considered to be an acceptable means of alignment. The use of the factors themselves made it difficult to separate out true differences due to instrument and reagent variation, from gross error. For most analytes, there is good discrimination between the COV and the affected population, so, whilst with the exception of MSUD, there is minimal risk of false negative results due to inaccuracy, this is not necessarily the case for false positive results. We believe the key contributors to inter-laboratory variation are instrument set up and internal standard.

The aim of this study was to investigate whether use of a common IS would reduce inter-laboratory variation in expanded NBS.

## 2. Materials and Methods

### 2.1. Preparation of Common Internal Standard Mix

l-phenylalanine (ring-D5, 98%), l-tyrosine (ring-D4, 98%), l-methionine (methyl-D3 98%), and l-leucine (5,5,5-D3, 99%) were obtained from CK Isotopes (Leicestershire, UK). l-carnitine:HCl, O-isovaleryl (N,N,N tri-methyl-D9, 98%), l-carnitine:HCl, O-octanoyl (N methyl-D3, 98%) and l-carnitine:HCl, O-decanoyl (N methyl-D3, 98%) were obtained from QMX Laboratories (Essex, UK). l-Glutarylcarnitine (N methyl-D3, 40%) was obtained from Solmedia (Shrewsbury, UK). Mixed acylcarnitine stable isotope stock standard solution (50 mL, 1 mM) was prepared in distilled water. The mixed stock standard was diluted 1:50 in distilled water to give final concentration of 20 μM. Aliquots (1.25 mL) were stored at −70 °C. Mixed amino acid stable isotope stock standard solution (500 mL, 2.5 mM) was prepared in distilled water. Aliquots (1.25 mL) were stored at −70 °C. C5DC stable isotope stock standard solution (250 mL, 5.91 mM) was prepared in distilled water. The stock standard was diluted 1:1000 in methanol to give final concentration of 5.91 μM. Aliquots (2.0 mL) were dried under nitrogen and stored at −70 °C. Aliquots of the three stable isotope stock standards were distributed to each laboratory by overnight courier on dry ice.

The common stable isotope IS mix was prepared locally by each laboratory as follows: Mixed amino acid stable isotope stock standard (1 mL) and mixed acylcarnitine stable isotope stock standard (1 mL) were added to a volumetric flask (500 mL) and 80% methanol (200 mL) was added. The C5DC stable isotope stock solution was reconstituted by the addition of 80% methanol (2–3 mL) to the vial. The solution was vortex mixed for 5 min prior to quantitative transfer of the contents to the volumetric flask containing the mixed acylcarnitine and amino acid stable isotopes. The volumetric flask was then made to the mark with 80% methanol to give final concentrations of 5, 0.04 and 0.024 μM, for the amino acids, acylcarnitines and glutarylcarnitine, respectively.

### 2.2. Study Design

Four screening laboratories participated in this study: South East Thames, West Midlands, Yorkshire and Wales. Each laboratory screened routine newborn blood spot samples using their in-house prepared stable isotope IS mix for a period of six months. Eight analytes were quantified by stable isotope dilution FIA–MS/MS and population data were collated centrally. Any laboratory specific “calibration factors” routinely used were included in the quantification. Each laboratory then screened routine newborn blood spot samples using a common stable isotope IS mix prepared centrally, for a period of six months. Population data were collated centrally and, importantly, any laboratory specific calibration factors used routinely with the in-house methods were removed. Approximately 102,000 babies were screened using in-house reagents (Wales *n* = 16,452, Yorkshire *n* = 21,121, South East Thames *n* = 28,843, West Midlands *n* = 35,515) and approximately 102,000 babies were screened using the common IS mix (Wales *n* = 16,326, Yorkshire *n* = 21,195, South East Thames *n* = 28,581, West Midlands *n* = 35,934).

### 2.3. Preparation of DBS Samples

Each of the four laboratories used the same methodology. A 3.2 mm sub-punch was taken from each DBS into a 96-well plate. The equivalent volume of blood in each sub-punch was assumed to be 3.1 µL. DBSs were extracted by addition of elution buffer (80% methanol, 150 µL) prior to agitating on a plate shaker for 20 min. Sample (10 µL) was introduced into the tandem mass spectrometer using variable flow injection analysis. Mobile phase consisted of 80% methanol containing 0.025% formic acid at an initial flow rate of 200 µL/min. The flow rate was decreased to 10 µL/min after 0.12 min and then increased to 600 µL/min at 1.1 min to flush out the ion source electrospray needle and the auto-sampler to ion source peek tubing. Data for quantification were acquired during the period of reduced flow rate.

### 2.4. Analysis of DBS Samples

Typical mass spectrometer settings were capillary voltage 3.5 kV, source block 120 °C, desolvation temperature 350 °C, desolvation gas flow, 800 L/h. Cone gas and collision energy were optimised for each analyte. Data were acquired by SRM using positive ionisation mode (leucine 132 > 86; methionine 153 > 107; C5 246 > 85; C5DC 276 > 85; phenylalanine 166 > 120; C8 288 > 85; tyrosine 182 > 136; C10 316 > 85). Quantification of each analyte was made by stable isotope internal calibration. The relative response ratio (RRR) of each analyte to its respective stable isotope IS was multiplied by the concentration of the appropriate stable isotope IS and then corrected for the nominal volume of blood in the 3.2 mm DBS sub punch, analyte concentration = RRR × 150/3.1) x concentration of IS. Analysis was performed on two Waters TQDs (Laboratory One), a Waters Xevo TQ and Waters Xevo TQD (Laboratory Two), a Waters Premier XE and Sciex API 4000 (Laboratory Three) and a Waters Premier XE and Waters TQD (Laboratory Four).

### 2.5. Statistical Analysis

Data analysis was performed using GraphPad Prism v7. Outliers were removed using the ROUT method (Q, false discovery rate = 1%) for each analyte. Summary statistics were determined; mean, relative standard deviation (RSD), coefficient variation (CV); 90th centile; coefficient of variation at the 90th centile. Results were summarised as Box and Whisker plots. The Horwitz equation [8] was used to predict the inter-laboratory variation for a given analyte. The Horwitz equation is independent of method, sample matrix and analyte and is based solely on the percentage purity of the analyte in the sample. The predicted relative standard deviation of reproducibility (PRSD) was calculated and used to predict the inter-laboratory variation for the eight analytes.

The data were also analysed using mixed effects models, in order to compare the intra- and inter-laboratory variability for the two types of IS. In the models, IS type was treated as a fixed effect and the intra- and inter-laboratory variances were included as random effects. It is the random effects that were of primary interest. In order to determine whether separate random effects were needed for the different IS types (which were used as an indicator for whether one type of IS was associated with better between-laboratory precision), the simplest model required to produce an adequate fit to the data was found for each analyte. This was conducted by using a standard model fitting process and the Akaike Information Criterion (AIC), with the model with the lowest AIC being selected. In most cases, the best model required separate between-laboratory variances to explain the data adequately, of which the smallest was associated with the common IS.

## 3. Results

**Figure 1:**
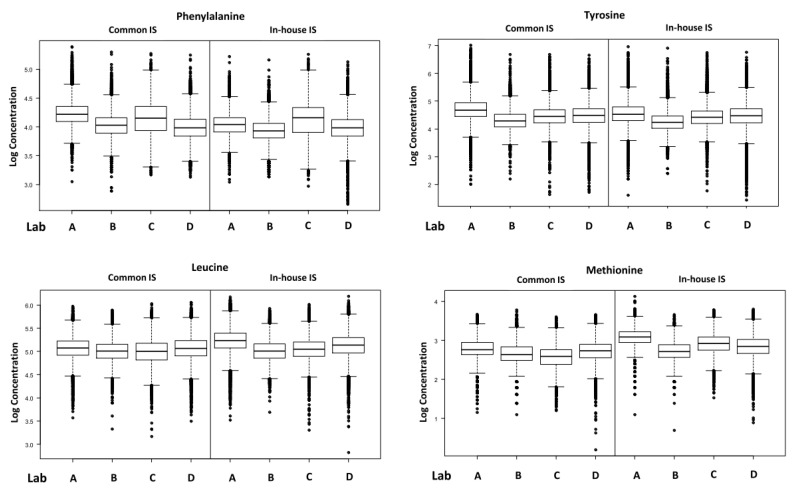
Box and whisker plots for the amino acids.

**Figure 2:**
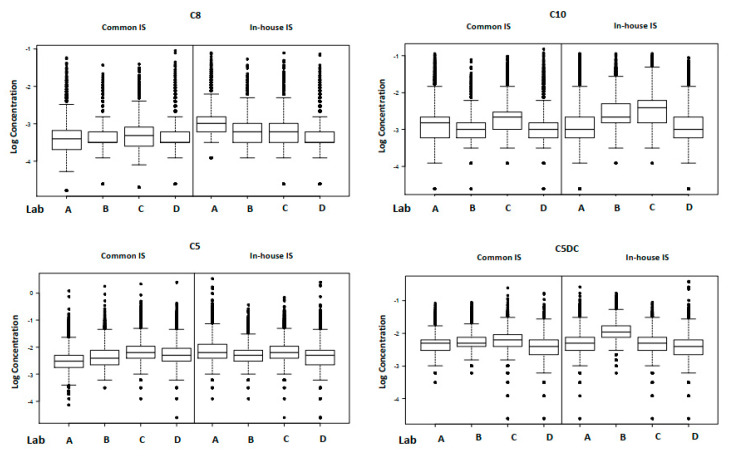
Box and whisker plots for the acylcarnitines.

Table 1 summarizes the mean, RSD and %CV at the 90th centile for each analyte, measured with both the individual laboratories in-house IS and with the common IS mix. Figure 1 and Figure 2 show the box and whisker plots for each analyte, measured using the individual laboratories in-house IS and with the common IS mix. Table 2 summarizes the inter-laboratory RSD, variance ratio and the intra-laboratory RSD from the linear mixed effect model. The intra-laboratory variances were not found to differ between the common IS and the in-house IS. In most cases, the best model required separate between-laboratory variances to explain the data adequately, of which the smallest was associated with the common IS. Variance ratios indicate the relative size of the between-laboratory variation for each analyte, with values above 1 showing that an improvement is observed when using the common IS. An improvement using the common IS was observed for total leucine, methionine, C5DC, C8 and C10 but not for phenylalanine, tyrosine or C5. The observed improvements were considered significant on the basis of whether separate random effects were required for the two IS types.

## 4. Discussion

Using the common IS mix significantly reduced inter-laboratory variation at the 90th centile for C5DC, C8, C10, total leucine and methionine. Using in-house IS, the average inter-laboratory variation at the 90th centile was 36.9% (range 20.5–60.1%) and this was reduced to 18.1% (range 10.6–30.8%) using the common IS. Conversely, using the common IS mix did not reduce the inter-laboratory variation for phenylalanine, tyrosine or C5. Using the in-house IS, the average inter-laboratory variation at the 90th centile for these three analytes was 29.4% (range 22.8–35.1%) and using the common IS it was 28.4% (range 21.6–35.0%). For C5, irrespective of the IS used, the inter-laboratory CV of the 90th centile remained >30%. However, Laboratory One was applying a “calibration factor” of 1.25 to their in-house results and this effectively masked the difference. Without the calibration factor, the true inter-laboratory variation at the 90th centile using the in-house IS mix would have been greater because the C5 results of the laboratory in question were negatively biased relative to the other three. Likewise, the same laboratory was applying calibration factors of 0.80 and 0.85 to the in-house phenylalanine and tyrosine results, respectively, and even with these factors, the laboratory had a positive bias relative to the other three. It can therefore be concluded that once the calibration factors have been accounted for, using a common IS mix does reduce the inter-laboratory variation at the 90th centile. Using a common IS mix removes the variation associated with the solid material (source, purity, choice of isotope) and the accuracy with which the stock IS and any subsequent dilutions are prepared. These factors contribute directly to the final concentration of the IS added to each DBS sub punch, and hence the reported result [9].

Interestingly, even when a common IS mix is used, the mean inter-laboratory variation at the 90th centile is still 21.9% (range 10.6% to 35.0%). The Horwitz equation [8] was used to calculate the PRSD, which is a predictor of inter-laboratory variation for a given analyte. The PRSD for leucine, methionine, phenylalanine, C5, C5DC, C8, tyrosine and C10 are 8.8, 10.9, 9.7, 17.3, 21.5, 20.8, 9.8 and 21.0% respectively, indicating that analytical performance of these analytes is still poorer than might be expected.

It is evident that factors other than the source of IS are also contributing to the variation in expanded NBS results. When quantitation is by internal calibration alone, the final result is influenced by the volume of blood assumed to be present in a 3.2 mm sub-punch, the extraction process and the instrument set up. In this study, the latter point was uncontrolled, as was the preparation of the working IS solution. Preparation of the working IS solution required the aliquots of IS mix to be reconstituted in a fixed volume of 80% methanol. This step, whilst critical, would be considered standard practice for a Clinical Laboratory accredited to the internationally recognized standard ISO 15189:2012 by the United Kingdom Accreditation Service (UKAS) and logic dictates that it would likely have been performed with a reasonable degree of accuracy, plus variation from this step would have contributed to the random effects of the linear mixed-effect model. Likewise, pre-analytical factors associated with DBS sampling such as haematocrit, spot size/volume and location of sub-punch can influence results [6], but again these were common to all laboratories. We hypothesize that variation in instrument set-up is also a significant variable, although we acknowledge that the presence of any interfering species from reagents, consumables, contamination or even the samples themselves would also have an effect.

## 5. Conclusions

For newborn screening programmes delivered by multiple laboratories using single analyte COVs, use of a common IS mix will significantly reduce inter-laboratory variation in expanded NBS. However, it is evident that other factors such as instrument set up are also contributing to analytical variation and it is important that laboratories aim to better understand and minimize these to ensure the quality, safety and efficacy of newborn screening programmes.

## Figures and Tables

**Table 1:** Summary of the mean, relative standard deviation and co-efficient of variation at the 90th centile for each analyte, measured with the individual laboratories in-house internal standard (IS) and with the common IS mix.

	In-House Internal Standard				Common Internal Standard			
Analyte	Number of Samples	Mean 90th Centile *	SD *	%CV	Number of Samples	Mean 90th Centile *	SD *	%CV
C5	99,317	0.07	0.02	30.5	100,952	0.06	0.02	35.0
C5DC	102,381	0.08	0.03	39.8	102,671	0.07	0.01	17.4
C8	97,269	0.03	0.01	29.7	100,924	0.02	0.00	10.6
C10	100,750	0.04	0.03	66.1	101,234	0.04	0.01	30.8
Leucine	102,078	122.4	25.1	20.5	102,484	114.2	14.5	12.7
Methionine	101,745	13.0	3.7	28.5	102,469	10.4	2.0	19.1
Phenylalanine	101,820	42.2	9.6	22.8	101,194	45.4	9.8	21.6
Tyrosine	99,889	53.1	18.9	35.1	100,067	58.3	16.7	28.6

* concentration in µmol/L.

**Table 2:** Summary of fitted intra- and inter-laboratory standard deviations obtained from the mixed models described in the text. Variance ratios indicate the relative size of the between-laboratory variation for each analyte (values above 1 show an improvement observed when using the common IS). The last column indicates whether observed improvements were significant; this was based on whether separate random effects were required for the two IS types. All standard deviations are shown in µmol/L.

	Inter Laboratory Standard Deviation				
Analyte	Common IS	In-House IS	Variance Ratio (in-House/Common IS)	Intra-Laboratory Standard Deviation	Improvement Observed?
C10	0.175	0.252	2.07	0.403	Yes
C5DC	0.082	0.199	5.86	0.276	Yes
C5	0.149	0.101	0.46	0.378	No
Leucine	0.040	0.098	5.93	0.246	Yes
Methionine	0.088	0.154	3.03	0.257	Yes
C8	0.053	0.163	9.59	0.359	Yes
Phenylalanine	0.107	0.079	0.55	0.223	No
Tyrosine	0.162	0.125	0.59	0.382	No
